# Hydrothermal Regime Variation and Ecological Effects on Fish Reproduction in the Yangtze River

**DOI:** 10.3390/ijerph182212039

**Published:** 2021-11-16

**Authors:** Wenxian Guo, Ning He, Gaofei Dou, Jianwen Hu, Hongxiang Wang

**Affiliations:** School of Water Conservancy, North China University of Water Resources and Electric Power, Zhengzhou 450046, China; guowenxian163@163.com (W.G.); hening077@163.com (N.H.); dougaofei2046@163.com (G.D.); hjw1141677044@163.com (J.H.)

**Keywords:** hydrothermal regime, water temperature, wavelet analysis, IHA-RVA method, ecological effects on fish

## Abstract

Water temperature, as one of the important water environment impact factors, has a significant impact on the survival and development of aquatic organisms. We selected water temperature data (1959–2017) from four key hydrological stations in the Yangtze River: Cuntan, Yichang, Hankou, and Datong. We analyzed the characteristics and variability of the Yangtze River hydrothermal regime by the Mann-Kendall method, wavelet analysis, and by the IHA-RVA method to analyze hydrothermal regime variations of the Yangtze River, and the response mechanisms of fish to variations in hydrothermal regimes were explored. The results show that (1) The annual average water temperature of the Yangtze River is warming, and Cuntan Station, Yichang Station, Hankou Station, and Datong Station have a sudden increase in temperature in 2002, 1995, 2003, and 2004, and the periodicity analysis demonstrates that Cuntan, Yichang, and Datong stations all have main periods of 24~32 years; (2) The overall variations of 32 hydrothermal indicators at Cuntan Station and Yichang Station reached 65% and 61%, which are close to the height variation; (3) With the construction of the Three Gorges Dam, the stagnant cooling effect caused the arrival date of the upper reproduction water temperature of “The four major fish species” to be delayed by about 23 days, and the stagnant heat effect caused the arrival date of the Chinese sturgeon reproduction upper limit water temperature to be delayed by about 30 days.

## 1. Introduction

Hydrothermal regime affects the energy exchange, metabolism, and habitat of biological communities (including plankton, benthic organisms, and fish) in river ecosystems, which restricts the healthy development of river ecosystems [1,2]. Water temperature is one of the important influencing factors of water enthusiasm. If it is too high or too low, it will threaten the safety of aquatic organisms and seriously affect the normal survival and reproduction of aquatic organisms [3]. The Yangtze River basin is one of the regions most affected by the anomalous temperature field of upper and high altitude in China, and it is also the birthplace of economic fish, such as “The four major fish species“, and the gene pool of fish in China [4], at the same time, the construction of the Three Gorges Dam (TGD) also affects the survival and reproduction of fish to a certain extent. Therefore, it is of great significance to carry out research on the ecological impact of variations in hydrothermal regime and fish reproduction for maintaining the health of the Yangtze River ecosystem.

Water temperature, as an important signal of hydrothermal regime, is directly reflects the thermal the river, which has an intensive impact on the physical and chemical of the river and directly or indirectly affect the whole life of aquatic organisms [5]. Analyzing the river water temperature variations and narrowing the adverse effects of river hydrothermal regime on fish through artificial control has historically been a research hot spot in the world [6]. Vliet explored the impact on fish habitats in different regions of the world based on hydrological, and water temperature modeling for future projections of global river flows and water temperatures with spreading several fish species and their maximum thermal tolerance and found the frequency and greatness of excessive maximum temperature toleration of selected fish species increased significantly [7]. Michel analyzed the effects of flow, precipitation, air temperature, and upstream lakes on river temperature and its temporary trends for devising stream temperature and flow in Switzerland over the last 50 years, showing that stream temperature trends have a serious impact on the spread of fish diseases, especially in lowland rivers, where the water temperature is more closely correlated with air temperature [8], Mariola Kędra Used linear trend analysis and complex wavelet transform to discover and assess the influence of water temperature variation causes on the natural hydrothermal regime of rivers, and analyzed daily river water temperature data for a period before and after modeling selected reservoirs in the Polish Carpathians, showing that weakening of natural air water temperature synchronization implies weakening of air temperature influence on river water temperature [9].

The aim of this paper is (1) to analyze the overall trend and periodic changes of the annual water temperature of the four representative hydrological stations in the Yangtze River Basin; (2) to analyze the impact of the Three Gorges Dam on the water temperature changes of Yichang station and Cuntan station; (3) Discuss the influence of the law of water temperature change in the long-term series and the construction status of the TGD on the reproduction time of fish. The results of this research can provide a scientific basis for the sustainable and healthy economic development and ecological restoration of the Yangtze River Economic Zone.

## 2. Materials and Methods

### 2.1. Study Area and Data Sources

The Yangtze River basin originates from the southwest side of the highest peak of the Qinghai Tibet plateau, Mount Tanggula, JiaoLandong Snow Mountain, to an place between 90°33′ and 122°25′ E and 24°30′ and 35°45′ N. Its total length is more than 6300 km, ranking third in the world after the Nile River in Africa and the Amazon River in South America in terms the world’s great rivers [10]. Its watershed covers an area of 1.8 million square kilometers, accounting for about 1/5 of the total land area of China [11]. It is a subtropical monsoon region with four distinct seasons, with an annual average temperature of 16 to 18 °C and a maximum variation of −4 to 40 °C. The lowest value the water temperature occurs from January to February, with the maximum daily variation of water temperature ranging from no more than 1 to 2 °C [12]. According to statistics, the total number of reservoirs include will in the joint scheduling of the Yangtze River basin in 2021 is 47, with a total regulating capacity of 106.6 billion cubic meters and a total flood control capacity of 69.5 billion cubic meters. Among them, 7 reservoirs of Three Gorges Group are included in the joint scheduling scope, with a total regulating capacity of 47.3 billion cubic meters and a total flood control capacity of 38.6 billion cubic meters, accounting for 44% and 56% of the total regulating capacity and flood control capacity of reservoirs in the Yangtze River basin joint scheduling statistics, 378 species of freshwater fishes in the Yangtze River basin, including 142 species of epidemic fishes, and 14 species of aquatic wildlife under the first and second national protection, many of which are unique to the Yangtze River [13].

**Figure 1:**
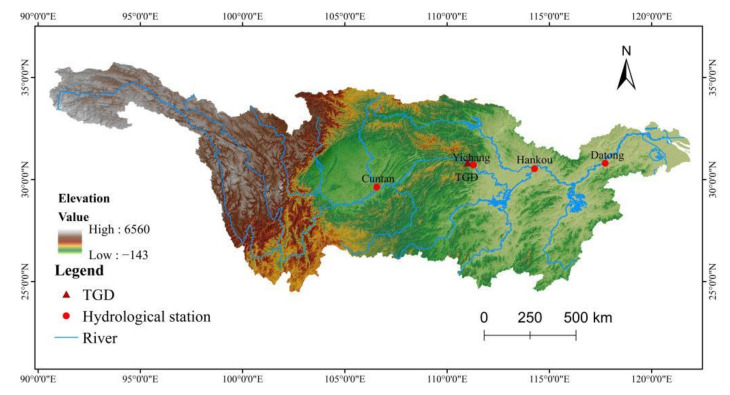
Location of the study region and the hydrological stations.

This paper chooses the long-term series of water temperature data from Cuntan, Yichang, Hankou, and Datong hydrological stations from the upstream to the downstream of the Yangtze River for research (Figure 1). Among them, Cuntan station is used as the control station of the Three Gorges into the reservoir and the upstream of the Yangtze River; Yichang station is the control station of the middle reaches of the Yangtze River and the TGD; Hankou station is near the confluence of the Han River and is an important control station of the confluence of the Han River; Datong station is used as the total control station of the of the Yangtze River [14], and linear interpolation method is used to interpolate the missing data. The average daily water temperature data come from the “Yangtze River Basin Hydrological Yearbook” (1960/1/1–2018/12/31) and Yangtze River Water Committee (CWRC) (http://www.cjw.gov.cn/, accessed on 12 November 2021).

### 2.2. Calculation Methods

#### 2.2.1. Analysis of the Average Annual Water Temperature Variation Trend and the Mutation Test

In this paper, the Mann-Kendall method(M-K) is used to test the trend of water temperature data. In the M-K non-parametric trend test, for a given significance level α (take 0.05 and 0.01), if the test statistic $\left|Z_{c}\right|\;\geq \;Z_{1-\frac{a}{2}}$, the null hypothesis is considered unacceptable [15]. The test for mutation points is first observed by the Mann-Kendall mutation test to discover whether the two curves UF, UB intersect at a given confidence level α, and then the mean difference *t*-test is used to further corroborate and discover whether the mutation year meets the mutability test need. For detailed steps refer to the literature [16].

#### 2.2.2. Analysis of the Annual Water Temperature Variation Period

Wavelet analysis can be used to qualitatively analyze the periodic variation of water temperature sequence, explore the variation characteristics of water temperature at different time scales, and estimate the changing trend of water temperature sequence by periodic transformation [17]. The following is the continuous wavelet transform:

(1) $$W_{f}\left(a,b\right)\;=\;{\left|a\right|}^{-\frac{1}{2}}\int f\left(t\right)\;\psi^{*}\;\left(\frac{t\;-\;b}{a}\right)\;dt$$

where $f\left(t\right)$ represents the original signal; $W_{f}\left(a,b\right)$ represents the wavelet coefficients; $f\left(t\right)$ represents the degree of correlation between the wavelet with a scale and displacement; $\psi \left(t\right)$ represents the mother wavelet function; a, b are the scale and translation; * is the conjugate.

In order to remove the false oscillation caused by the real wavelet coefficient, the complex Morlet wavelet is selected as the wave in this paper, and its formula is expressed.

(2) $$\psi_{0}\left(t\right)\;=\;\frac{1}{\sqrt{\pi f_{b}}}\;\exp\;\left[-\frac{t^{2}}{f_{b}}\;+\;\left(2\pi f_{c}t\right)\;i\right]$$

where $f_{b}$, $f_{c}$ are wavelet bandwidth and wavelet center frequency, respectively; i is the symbol of the imaginative number.

#### 2.2.3. Analysis of the Degree of Water Temperature Variation

Range of Variability Approach and indicator of Hydrologic Alteration was selected to establish the evaluation of ecological hydrological index and quantify the variation degree of river hydrological regime [16,17], calculated by the following equation:

(3) $$D_{i}\;=\;\left|\frac{N_{oi}-N_{e}}{N_{e}}\right|\;\times \;100\%$$

where $D_{i}$ is the degree of hydrological alteration of the ith IHA indicator; $N_{oi}$ is the number of years in which the ith IHA falls within the RVA target threshold of the observed years after the disturbance; $N_{e}$ is the number of years in which the changed IHA indicator is expected to fall within the RVA target, which can be assessed by $r\cdot N_{T}$, and r is the proportion of the IHA falling within the RVA target threshold before the disturbance.

The hydrological variation degree of a single indicator cannot reflect the overall degree of variation, so this article uses the overall hydrological variation degree to reflect the overall variation of water temperature. The calculation principle is detailed in literature [18], and the calculation is as follows:

(4) $$D_{0}\;=\;{\left(\frac{1}{n}\sum_{i=1}^{n}D_{i}^{2}\right)}^{0.5}$$

where n is the number of indicator and defined $D_{0}$ values between 0–33% as low alteration; 33–67% as moderate alteration; and 67–100% as high alteration.

## 3. Results

### 3.1. Temporal Spatial Evolution Characteristics of the Yangtze River Hydrothermal Regime

#### 3.1.1. Hydrothermal Area and Interannual Distribution Characteristics

**Figure 2:**
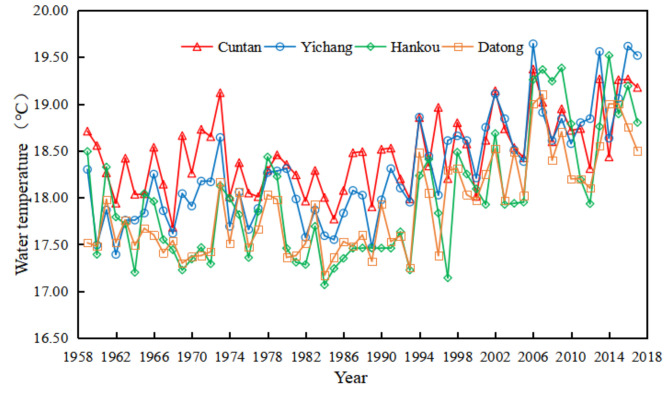
Annual average water temperature variation of the Yangtze River.

**Figure 3:**
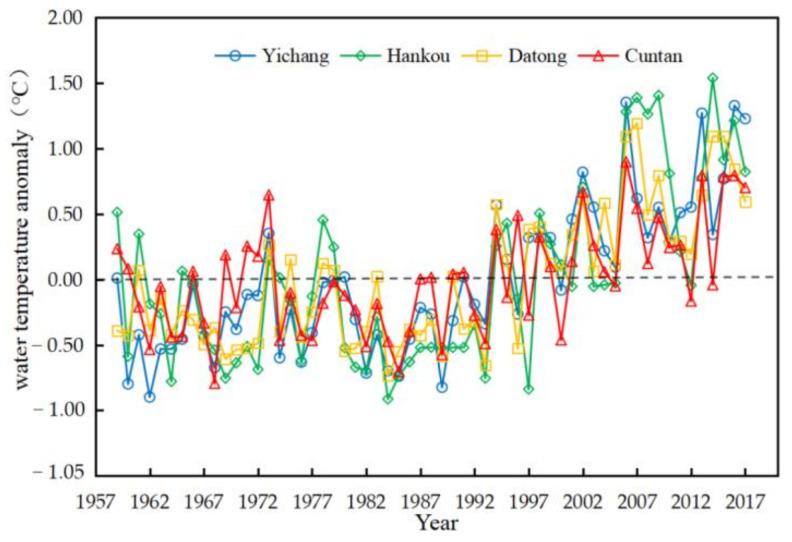
Temperature anomaly diagram of the Yangtze River.

The reservoir has huge heat stagnation, heat dissipation, and heat release, and the range of water temperature variation in the lower reaches of the reservoir is higher than in Hankou [19]. The average annual water temperature of Cuntan Station, Yichang Station, Hankou Station, and Datong Station of the of the Yangtze River shows an overall warming trend (Figure 2). The average annual water temperature of Cuntan Station, Yichang Station, Hankou Station, and Datong Station are, Respectively 18.47 °C, 18.29 °C, 17.98 °C, 17.91 °C. The water temperature difference at the four stations was negative before 1994, except for some positive values in some years, indicating that the water temperature of the Yangtze River was at a relatively low level before 1994. After 1994, Cuntan Station, Yichang Station, Hankou Station, and Datong Station showed different heating patterns. Especially after entering the 21st century, the average water temperature moment was positive, and the water temperature increased substantially (Figure 3).

#### 3.1.2. Trend and Sudden Variation Characteristics of Hydrothermal Regime

The M-K non-parametric trend test was conducted for the annual water temperature at four hydrological stations on the of the Yangtze River (Table 1), and *t*-test was used to corroborate the results of M-K variation test (Table 2). The results show that the water temperatures of the main hydrological stations of the Yangtze River presents a significant warming trend, and through comparative analysis, it was found the water temperature at Cuntan showed abrupt points in 2002, Yichang in 1995, and Hankou and Datong in 2003 and 2004, among them, Yichang station has the earliest abrupt points in water temperature, and the other three stations have abrupt points around 2003, which may be affected by multiple factors such as the climate and the operation of the Gezhouba Dam and the TGD. Although the water temperature at Yichang Station had abrupt points before the construction of the TGD in 2003 (1995), the influence was relatively small compared with the stagnation heat caused by the impoundment of the Three Gorges project [20].

#### 3.1.3. Hydrothermal Regime Periodic Variation Characteristics

**Figure 4:**
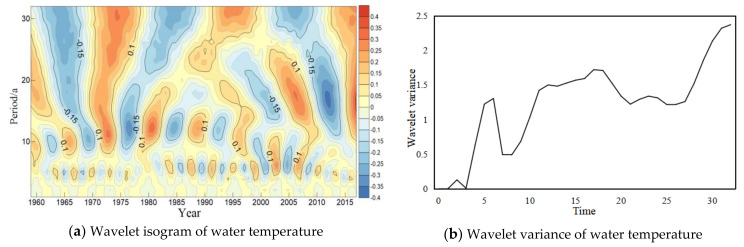
Wavelet isogram (**a**) and wavelet variance map (**b**) of water temperature at Cuntan station.

**Figure 5:**
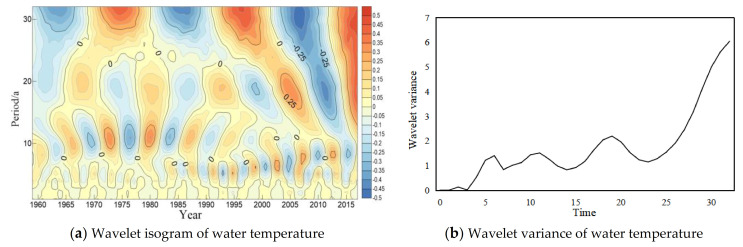
Wavelet isogram (**a**) and wavelet variance map (**b**) of water temperature at Yichang station.

**Figure 6:**
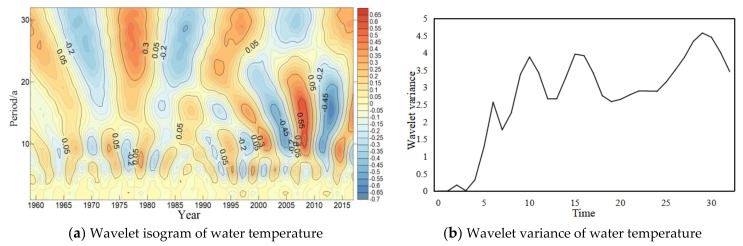
Wavelet isogram (**a**) and wavelet variance map (**b**) of water temperature at Hankou station.

**Figure 7:**
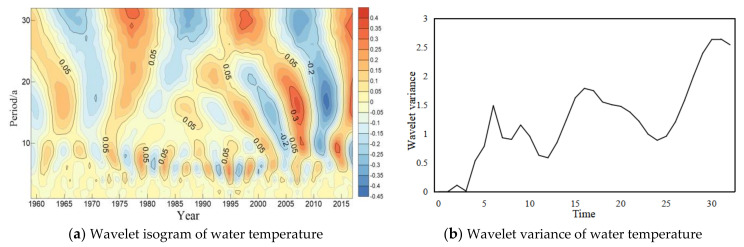
Wavelet isogram (**a**) and wavelet variance map (**b**) of water temperature at Datong station.

The wavelet analysis method was used to analyze trend of the periodic variation of water temperature in long-term scale at four major hydrological stations of the Yangtze River (Figure 4, Figure 5, Figure 6 and Figure 7). In general, four hydrology stations have significant variations throughout the time domain. four scales of periodic variation exist at Cuntan, Yichang, and Hankou stations, while three scales of periodic variation exist at Datong station. Among them, four more obvious peaks in the Cuntan station wavelet variance of water temperature, and the largest peak agrees to a time scale of 19 years, the periodic oscillation is the strongest around 19 years, which is the first main periodic of water temperature variation at Cuntan station (Figure 4b); There are five obvious peaks in the wavelet variance of water temperature at Yichang station, and the largest peak agrees to a time scale of 32 years, that is, the periodic oscillation is the strongest around 32 years, which is the first main cycle of water temperature variation at Yichang station (Figure 5b);The wavelet variance of water temperature of Hankou and Datong stations both have five more obvious peaks, and the maximum peak agrees to a time scale of 29 years, that is, the periodic oscillation is strongest around 29 years, the first main periodic of water temperature variation at both stations (Figure 6b and Figure 7b).

#### 3.1.4. Characteristics of Hydrothermal Variability Variation Degree

Given the serious lack of day-by-day data in Hankou and Datong, this paper uses the RVA method to Calculate the number of years that 32 parameters fall within the target range based on the more complete day-by-day water temperature data from 1993–2017 at the upstream Cuntan station and 1983–2017 at the midstream Yichang station, as a basis for variability analysis and to quantify the degree of variability of each water temperature indicators at the two stations (Table 3 and Table 4). The comprehensive results show that, among 32 indicators at Cuntan station, the indicator with moderate variations account for 40% of the total indicator, and those with high variations account for 60% of the total indicator; the overall degree of variation of the indicators reached 65.00%, the close to the degree of high variation. Among the 32 indicators in Yichang station, the indicator of moderate variation accounted for 50% of the total, the indicators of high variation accounted for 31.25% of the total, and the indicator of low variation accounted for 18.75% of the total; the overall degree of variation of the indicator reached 61.02%, close to the degree of high variation (Table 5), indicating the water temperature in Yichang station was significant. There were obvious variations around 2003.

### 3.2. Effects of Variations in Hydrothermal Regime on Fish

Fish reproduction is driven by water temperature, and proper water temperature is a necessary regime for maturing fish gonads, and an important guarantee for the smooth hatching of fish eggs and embryos [21]. The middle and lower reaches of the Yangtze River are important breeding areas for “The four major fish species” and Chinese sturgeon, but, with the influence of human climate variation, the fry runoff of “The four major fish species” and Chinese sturgeon have showed a decreasing trend. According to relevant research, the reproduction of “The four major fish species” is mainly from April to June each year, and the suitable water temperature for reproduction is 21~24 °C. when the water temperature is lower than 18 °C, no reproduction occurs is found [22]. The reproduction period of Chinese sturgeon is mainly from in October to November every year, the suitable water temperature for reproduction is 17–20.2 °C, and the average water temperature for multiple reproduction is 18.6 °C. Beyond this range, the reproduction frequency will decrease significantly. So far, no records of natural reproduction of Chinese sturgeon have been found when the water temperature is lower than 15.0 °C or higher than 21.0 °C [23]. According to the study on the effect of the Yangtze River water temperature on fish reproduction, it can be found the reservoir storage and dam effect and the discharge water temperature will have a direct impact on the survival, and reproduction of river aquatic organisms, which may force the fish reproduction period the appropriate water temperature, so it has been an important issue of widespread concern in river management [24].

The data of “The four major fish species” and Chinese Sturgeon came from the “Ecological Environment Monitoring Bulletin of the Three Gorges Project and the Survey Manual of Fishery Natural Resources in Inland Waters”, and were investigated by referring to literature of relevant scholars [25,26,27,28].

#### 3.2.1. Effects on Reproduction of “The Four Major Fish Species”

“The four major fish species” were the general term for *Mylopharyngodonpiceus*, *Ctenopharyngodonidella*, *Hypophthalmichthysmolitrix*, and *Aristichthysnobilis*, which belong to the family *Cyprinidae*. the fishes are epidemic economic fish in China, and were also the cornerstone of Chinese freshwater fisheries. “The four major fish species” began to spawn in late April in the 1960s. the first reproduction time before the impoundment of the Three Gorges Reservoir (TGR) is from the end of April to the beginning of May, compared to this, the reproduction time have little variation after the impoundment of the TGR, there were no reproduction in April. After impounding in 2003, the temperature reached 18 °C at the end of April 2005 and 2006. “The four major fish species” reproduction water temperature of 18 °C was postponed to May, and no reproduction behavior was detected in April [29]. In 2009, the reproduction time was 9 May, and the reproduction temperature was 18.0 °C; the first reproduction time in 2011 was 18 June, and the reproduction temperature was 23.8 °C; the first reproduction time in 2013 was 13 May, the reproduction temperature were 19.4 °C; the first reproduction time in 2015 were delayed later, 25 May, and the reproduction temperature were 20.8 °C; the first reproduction time in 2016 was delayed even later, May 26, the reproduction temperature were 20.4 °C. It follows that the reproduction time was postponed from May 8 (average value) before the water storage to May 26, which was delayed by 18 days. From the analysis of the reproduction time of “The four major fish species” in recent years, it can be seen that from April to July before the impoundment of the reservoir is been gradually compressed to May to June after the impoundment, the reproduction cycle is gradually shortened from 60 to 80 days to 20 to 50 days. The law of variation is closely related to the delay of water temperature reaching 18 °C.

To further analyze the water and heat of “The four major fish species”, a characteristic year with water temperature in May before and after the impoundment of the TGD in 2003 was selected. And based as well on Cai’s conclusion that “The four major fish species” lay eggs only when the reproduction water temperature is greater than 18 °C during the breeding season. Take the date when “The four major fish species” meet the daily average water temperature to reach 18 °C for the first time as the starting point for the study, Further, draw that the dates for meeting the lower limit of the breeding water temperature of “The four major fish species” are postponed by 25 days, 16 days, and 28 days (Table 6). “The four major fish species” are significantly affected by the cold stagnation effect after water storage, which delays the lower limit of reproduction water temperature. This conclusion is consistent with the conclusion of Mao‘s “The impact of the cold stagnation effect of the reservoir during the reproduction “The four major fish species“ on the downstream water temperature” [30].

#### 3.2.2. Effects on Reproduction of Chinese Sturgeon

**Figure 8:**
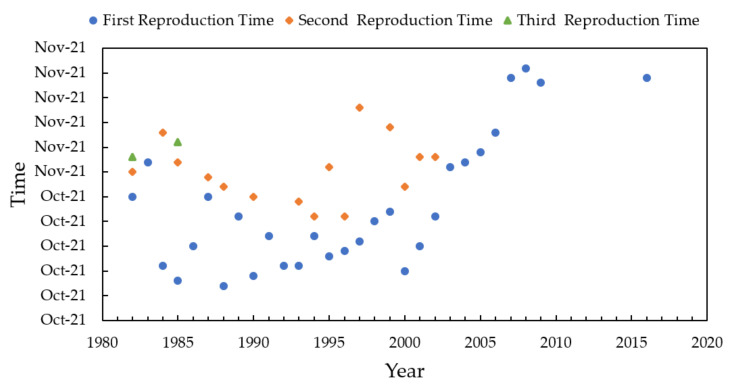
Variation of reproduction time of Chinese sturgeon in the Yangtze River.

Chinese sturgeon, a fish of the sturgeon family of the *Acipenseridae*, is one of the key protected wild animals in China, and is the largest fish in the Yangtze River. Based on the analysis of reproduction Chinese sturgeon from October to November, the average water temperatures in October and November was 18.4 °C and 18.3 °C after the impoundment of Gezhouba Reservoir and before the impoundment of TGD (1983–2002). The multi-year average water temperatures in October and November after the impounding of TGD (2003–2017) was 19.1 °C and 19.0 °C, the results showed the average temperature of the Yangtze River increased by about 1 °C compared with that before water storage (1983–2002), which met the water temperature regime for the breeding of Chinese sturgeon. In the 1980s, the first reproduction date of Chinese sturgeon was mostly concentrated in mid-October to early November each year; in the 1990s, the first production date was concentrated in mid late October, which was, overall, earlier than in the 1980s; while after entering the 21st century, especially after 2003, the first reproduction date of Chinese sturgeon was significantly delayed due to the storage of water in Three Gorges, and after 2003, the first reproduction dates of Chinese sturgeon were all after 2003, the first reproduction date of Chinese sturgeon fell into November, and after 2006, it was even delayed to late November so that its reproduction date was postponed by nearly one month (Figure 8).

To further explore the reproduction time of Chinese sturgeon after the Three Gorges impoundment, a characteristic year with higher water temperature in October before and after TGD impoundment in 2003 was selected for hydrothermal analysis of Chinese sturgeon. According to statistics, Chinese sturgeon spawned 48 times from 1982 to 2012 at Yichang Station, and then spawned only once in 2016, so the first reproduction time of Chinese sturgeon was used for the hydrothermal analysis, as can be seen from Table 7, the average water temperature in October of the three characteristic years of the first reproduction time of Chinese sturgeon breeding were 19.6 °C and after water storage were 23.1 °C, which was 3.5 °C higher than that before water storage, and the average water temperature in November of the three characteristic years before the water storage was 16.8 °C, and after water storage was 20.1 °C, which was 3.3 °C higher than before water storage. The average water temperature in November of the three characteristic years was 16.8 °C and 20.1 °C after impoundment, which was 3.3 °C higher than that before impoundment. The first reproduction time of Chinese sturgeon was delayed by 22 days, 34 days, and 34 days in different characteristic years before and after water storage, indicating that the reproduction of Chinese sturgeon was significantly affected by the stagnant heat effect after water storage, resulting in the trend of delaying the date of reaching the upper reproduction water temperature [31]. This conclusion is consistent with Zhang’s “Study on eco-hydrological Effects of Damming river in the middle and lower reaches of Yangtze River”.

## 4. Discussion

The water temperature of the Yangtze River is mainly affected by climate variation and humans, and the main human is the “Stagnant heat and cold“ effect caused by the impounding of the Three Gorges and other large reservoirs. Studies show that other mortal such as agriculture, animal cultivation, vegetation decrease, river widening, and afforestation, will lead to significant variations in river temperature [32]. Considering the hydrological background of the Yangtze River basin, it is not difficult to speculate on the main causing the abrupt of water temperature in the Yichang reach, It is probably related to the impounding of the TGR [33]. “The four major fish species” spawn in the warming period from April to June under natural regime and never reproduce when the water temperature is below 18 °C. Peng found that the water temperature below the Three Gorges Dam reached 18 °C, and the reproduction time of “The four major fish species” was delayed, especially after the storage level reached 156 m and 175 m, and the first detection of The fry “The four major fish species” below the dam was delayed by more than one month compared with the crisis [34]. Chang Tao showed that, due to the huge storage capacity of the TGD, the water temperature in the downstream runoff showed stagnant phenomenon (from October to the next January), resulting in variations in the water temperature pattern of reproduction grounds and a delay in occurring the upper limit of breeding water temperature (20 °C), which was speculated to be one of the factors causing abnormal breeding activities of Chinese sturgeons [35]. The thermal retention effect of the upstream reservoir on Cuntan and Yichang stations indicates the average water temperature decreases in spring and summer (March–June), while the average water temperature increases significantly in autumn and winter (July–February of the following year), which will delay and shorten the reproductive, the annual extreme water temperature of Cuntan and Yichang stations have different degrees of delay in appearance compared with the natural period, and the base temperature index has increased, showing the heat retention effect of the upstream reservoir is obvious, especially the variation in the number of reversals directly affect the living environment of aquatic plants and animals, hinder the growth aquatic plants and animals, and will adversely affect the habitat of aquatic organisms and reproduction of aquatic organisms such as fish, thus affecting the river ecosystem stability.

At present, Yangtze River hydrothermal form is still at the stage of theoretical exploration. Some scholars proposed to control the excessively high-water temperature at the downstream inlet of the TGD by arranging large-scale surface photovoltaics (PPV) in the terrace reservoirs of the dry and hot valleys to block solar radiation and shortwave radiation [18], however, it is difficult and unrealistic to regulate the water temperature of the Yangtze River with engineering measures. Increasing vegetation cover in the upstream hot valley can be used to delay the continuous rise of the water temperature of the TGD or adjusting the flood dismiss of the reservoir to adjust the water temperature of the target fish reproduction period, to reduce the influence of the reservoir “Stagnant heat and cold” on the fish. For example, for “The four major fish species”, the water temperature during the reproduction period from April to June can be adjusted so that the date when it reaches above 18 °C will not be shortened. For the Chinese sturgeon, the water temperature from October to November can be adjusted to spawn. The suitable range of the field is 17–20.2 °C. It should be pointed out that although the influence of water temperature in other time periods on the runoff of fry of “The four major fish species” is not as intuitive as that in the reproduction period, the influence of water temperature in the non-reproduction period on the number, development, maturity, and activity of parents will eventually affect their reproduction. Therefore, the water temperature should be considering as an important ecological factor in the environmental regulation of the Three Gorges, so as to meet the ecological warming of the target fish.

## 5. Conclusions

Analyzing the variation of water enthusiasm in the Yangtze River Basin and its ecological impact on fish reproduction has important ecological significance for the health protection of the Yangtze River and lakes. The annual mean water temperature at four representative hydrographic stations on the Yangtze River has shown a warming trend over the last 60 years, and the abrupt years of Cuntan Station, Yichang Station, Hankou Station, and Datong Station are 2002, 1995, 2003, and 2004, respectively, and the main periods of Cuntan Station, Yichang Station, and Datong station are all 24 to 32 years. However, the storage of water in the Three Gorges Reservoir has caused variations in the ecological environment of the river section below the dam, and at the same time has seriously affected fish reproduction, resulting in delayed reproduction time and reduced spawning frequency, which fully illustrates the seriousness of the impact of the storage of water in the TGR on fish reproduction in the river section below the dam.

## Figures and Tables

**Table 1:** Results of M-K analysis of the flow and water temperature variation trend at the main hydrology stations in the Yangtze River.

Hydrological Station		Cuntan	Yichang	Hankou	Datong
water temperature	Test discriminant	$\left|Z_{c}\;>2.58\right|$	$\left|Z_{c}\;>2.58\right|$	$\left|Z_{c}\;>2.58\right|$	$\left|Z_{c}\;>2.58\right|$
		Significant warming	Significant warming	Significant warming	Significant warming

**Table 2:** The *t*-test of annual temperature.

Hydrological Station	Category	Mutation Year	Base Year	Mutation Index AI	Statistic
Cuntan	Water temperature	2002	43	0.72	4.63
Yichang	Water temperature	1995	36	1.12	8.35
Hankou	Water temperature	2003	49	1.13	7.61
Datong	Water temperature	2004	49	1.07	7.18

**Table 3:** Results of the indicator of Water Temperature Variation analysis for Cuntan Station.

Hydrological Indicator	Before Mutation	After Mutation	Degree of Variation (%)	Hydrological Indicator	Before Mutation	After Mutation	Degree of Variation (%)
Average water temperature in January (°C)	10.25	11.1	−68	Annual average 90-day minimum water temperature (°C)	11.94	12.7	12.7
Average water temperature in February (°C)	11.2	11.9	11	Annual average 1-day maximum water temperature (°C)	26.25	26.8	26.8
Average water temperature in March (°C)	14.85	14.3	49	Annual average 3-day maximum water temperature (°C)	26.25	26.47	26.47
Average water temperature in April (°C)	18.8	18.4	27	Annual average 7-day maximum water temperature (°C)	26.21	26.17	26.17
Average water temperature in May (°C)	22.05	22	−11	Annual average 30-day maximum water temperature (°C)	25.43	25.49	25.49
Average water temperature in June (°C)	23.5	23.2	9	Annual average 90-day maximum water temperature (°C)	24.42	24.62	24.62
Average water temperature in July (°C)	24.25	24.6	49	River Basal Temperature Index	0.497	0.542	−82
Average water temperature in August (°C)	25.1	25.2	−46	Time of occurrence of annual minimum (d)	11	18	−52
Average water temperature in September (°C)	22.1	22.9	−49	Time of occurrence of annual maximum (d)	214	227	−68
Average water temperature in October (°C)	19.2	20.6	−46	Number of low water temperature pulses (times)	1	2	−34
Average water temperature in November (°C)	16.1	17.5	−64	Low water temperature pulse duration (d)	80	37.5	−32
Average water temperature in December (°C)	12.15	13.5	−49	Number of highwater temperature pulses (times)	2	5	−100
Annual average 1-day minimum water temperature (°C)	9.25	9.9	9.9	High water temperature pulse duration (d)	46	5	−100
Annual average 3-day minimum water temperature (°C)	9.283	10.3	10.3	Average annual rate of water temperature rise	0.975	0.2	−100
Annual average 7-day minimum water temperature (°C)	9.336	10.43	10.43	Average annual rate of water temperature decline	−0.8	−0.2	−100
Annual average 30-day minimum water temperature (°C)	9.897	10.82	10.82	Number of water temperature reversals per year (times)	12	106	−100

**Table 4:** Results of the indicator of Water Temperature Alteration analysis for Cuntan Station.

Hydrological Indicator	Before Mutation	After Mutation	Degree of Variation (%)	Hydrological Indicator	Before Mutation	After Mutation	Degree of Variation (%)
Average water temperature in January (°C)	10.2	13.8	−100	Annual average 90-day minimum water temperature (°C)	10.92	12.14	−33.33
Average water temperature in February (°C)	10.05	12.05	−85.19	Annual average 1-day maximum water temperature (°C)	27	27	33.33
Average water temperature in March (°C)	12.65	12	−33.33	Annual average 3-day maximum water temperature (°C)	26.87	26.93	66.67
Average water temperature in April (°C)	16.98	14.5	−100	Annual average 7-day maximum water temperature (°C)	26.72	26.79	66.67
Average water temperature in May (°C)	21.5	19.4	−33.33	Annual average 30-day maximum water temperature (°C)	26.11	27.07	33.33
Average water temperature in June (°C)	23.68	22.95	−6.667	Annual average 90-day maximum water temperature (°C)	24.9	24.89	33.33
Average water temperature in July (°C)	24.25	24.6	−25.93	River Basal Temperature Index	0.5021	0.5855	−66.67
Average water temperature in August (°C)	26	25.6	66.67	Time of occurrence of annual minimum (d)	30	59	−66.67
Average water temperature in September (°C)	23.13	24.35	3.704	Time of occurrence of annual maximum (d)	214	227	−68
Average water temperature in October (°C)	20.2	22.1	−70.37	Number of low water temperature pulses (times)	2	2	−20
Average water temperature in November (°C)	16.95	19.2	−100	Low water temperature pulse duration (d)	54	24.75	−33.33
Average water temperature in December (°C)	12.65	16.8	−100	Number of highwater temperature pulses (times)	3.5	2	−48.72
Annual average 1-day minimum water temperature (°C)	9.2	10.8	−83.33	High water temperature pulse duration (d)	12	44	−50
Annual average 3-day minimum water temperature (°C)	9.217	10.8	−83.33	Average annual rate of water temperature rise	0.2	0.2	−33.33
Annual average 7-day minimum water temperature (°C)	9.314	10.94	−83.33	Average annual rate of water temperature decline	−0.2	−0.2	−33.33
Annual average 30-day minimum water temperature (°C)	9.662	11.3	−83.33	Number of water temperature reversals per year (times)	112	103	−16.67

**Table 5:** Overall hydrological variability.

Hydrological Alteration of Each Group/%.						Overall Hydrological Variation/%
Station	Group 1	Group 2	Group 3	Group 4	Group 5	
Cuntan	44.35	69.37	60.61	74.55	100.00	65.00
Yichang	70.48	64.35	50.58	39.95	28.87	61.02

**Table 6:** “The four major fish species” breeding situation.

	Characteristic Year		Mean Water Temperature in May of the Characteristic Year (°C)	Mean Water Temperature in June of the Characteristic Year (°C)	Mean Water Temperature in July of the Characteristic Year (°C)	The Average Daily Water Temperature Reached 18 °C Data for the First Time	Delay Time (Days)
Before water storage	Dry year	1984	20.9	23.8	24.7	17 April	
	Normal year	1983				23 April	
	Wet year	2002				11 April	
After water storage	Dry year	2013	19.3	19.3	23.0	12 May	25
	Normal year	2009				9 May	16
	Wet year	2012				9 May	28

**Table 7:** Chinese sturgeon breeding situation.

	Characteristic Year		Average Water Temperature in October (°C)	Average Water Temperature in November (°C)	Mean Water Temperature in October of the Characteristic Year (°C)	Mean Water Temperature in November of the Characteristic Year (°C)	Time of First Reproduction	Delay Time (Days)
Before water storage	Dry year	1986	18.9	15.6	19.6	16.8	21 October	
	Normal year	1995	20.3	17.5			19 October	
	Wet year	1994	19.7	17.1			23 October	
After water storage	Dry year	2006	22.8	20.2	23.1	20.1	13 November	22
	Normal year	2009	23.0	19.4			23 November	34
	Wet year	2016	23.5	20.7			24 November	34

## Data Availability

Not applicable.

## References

[1] Ducharne A. (2008). Importance of stream temperature to climate change impact on water quality. Hydrol. Earth Syst. Sci..

[2] Caissie D. (2006). The thermal regime of rivers: A review. Freshw. Biol..

[3] Liu M., Wu Z., He J., Shen X., Gao Y., Yu Z. (2014). Thermodynamics and stratification in Xin’anjiang Reservoir (Lake Qiandao). J. Lake Sci..

[4] Yang G., Xu X. (2020). Foundation and Strategy of Well-Coordinated Environmental Conservation and Avoiding Excessive Development in the Yangtze River Economic Belt. Bull. Chin. Acad. Sci..

[5] Mao J., Dai H. (2016). Effects of large water conservancy and hydropower projects on major aquatic species and regu-lation. J. Hohai Univ..

[6] Olson P.A., Foster R.F. (2011). Temperature Tolerance of Eggs and Young of Columbia River Chinook Salmon. Trans. Am. Fish. Soc..

[7] Vliet M.T.H., Ludwig F., Kabat P. (2013). Global streamflow and thermal habitats of freshwater fishes under climate change. Clim. Chang..

[8] Michel A., Brauchli T., Lehning M., Schaefli B., Huwald H. (2020). Stream temperature and discharge evolution in Switzerland over the last 50 years: Annual and seasonal behaviour. Hydrol. Earth Syst. Sci..

[9] Kędra M., Wiejaczka A. (2018). Climatic and dam-induced impacts on river water temperature: Assessment and management im-plications. Sci. Total. Environ..

[10] Lin Z., Levy J.K., Xu X., Zhao S., Hartmann J. (2005). Weather and seasonal climate prediction for flood planning in the Yangtze River Basin. Stoch. Environ. Res. Risk Assess..

[11] Zhang P., Zhu X., He Q. (2020). Spatial-temporal differentiation and balance pattern of supply and demand of ecosystem services of the Yangtze River Economic Zone. Ecol. Sci..

[12] Mao H., Wang Z., Lin R., Zou M. (2019). Analysis of water-sediment characteristics and influence factors of upper and lower river reaches before and after impoundment of Three Gorges Reservoir. J. Water Resour. Water Eng..

[13] Tali P.A., Bhat M.M., Lone F.A. (2021). Seasonal Spatio-Temporal Variability in Temperature over North Kashmir Himalayas Using Sen Slope and Mann-Kendall Test. J. Climatol. Weather Forecast.

[14] Hu G., Song H. (2012). Analysis of Air-Temperature Variation Trend and Abrupt Change in Jinan Based on Mann-Kendall Test. J. Univ. Jinan.

[15] Sang Y., Wang Z., Liu C. (2013). Applications of wavelet analysis to hydrology: Status and prospects. Prog. Geogr..

[16] Zheng X., Yang T., Cui T., Xu C., Zhou X., Li Z., Shi P., Qin Y. (2020). A revised range of variability approach considering the morphological alteration of hydrological indicators. Stoch. Environ. Res. Risk Assess..

[17] Gao Y., Vogel R.M., Kroll C.N., Poff N.L., Olden J.D. (2009). Development of representative indicators of hydrologic alteration. J. Hydrol..

[18] Wang H., Zha H., Zhuo Z., Qian Z., Guo W. (2019). The evaluation of hydrological regime in Four River Basins based on IHA-RVA method. J. China Inst. Water Resour. Hydropower Res..

[19] Zhou J., Yang Q., Zhang M. (2019). Thermal-effect of the upper Yangtze reservoirs and countermeasures. J. Lake Sci..

[20] Yu W., Xia Z., Yu G., Cai Y. (2007). Water temperature variation in Three-Gorges Reservoir and its influence on procreation of Chinese sturgeon. J. Hohai Univ..

[21] Chen J., Li Q. (2015). Assessment of Eco-operation Effect of Three Gorges Reservoir During Trial Run Period. J. Yangtze River Sci. Res. Inst..

[22] We Q. (2020). Conservation of Chinese sturgeon (Acipenser sinensis) based on its life history: Dilemma and breakthrough. J. Lake Sci..

[23] Chen Q., Zhang J., Kangle M., Chen Y., Guan T., Wang G., Lin Y. (2020). Effects of hydropower development on aquatic eco-environment and adaptive managements. Adv. Water Sci..

[24] Liao X., Zhu B., Chang J. (2017). Study on conservation of Chinese sturgeon. Yangtze River.

[25] Sun L., Zhao F., Zhang T., Zhuang P. (2017). Research progress on resources and genetic diversity of Chinese sturgeon. Fish. Inf. Strategy.

[26] Guo W., Xia Z., Wang H., Zhang Y. (2008). Multiple scale analysis on water temperature variation of Yichang Hydrological Station in recent 50 years. J. Hydraul. Eng..

[27] Lin G. (1982). A survey on the spawning grounds of the "four famous Chinese carps" in the Yangtez river after dammed by the key water control project at Gezhouba. J. Fish. China.

[28] Yi B. (1958). Reproductive protection of freshwater fish resources. China Fish..

[29] Cai Y., Yang Z., Wei X. (2017). Effect of Water Temperature Variation After Impoundment of the Three Gorges Dam on Natural Reproduction of the Four Major Chinese Carps. Adv. Eng. Sci..

[30] Mao J., Hui E. (2020). Influence of cold-temperature stagnation phenomenon induced by reservoir impoundment on downstream water temperature in spawning period of four major Chinese carps. J. Drain. Irrig. Mach. Eng..

[31] Chen C., Li M., Gao X., Jiang W., Liu H., Duan Z., Cao W. (2020). The status of the early-stage fish resources and hydrologic influencing conditions in the Yichang section in the middle reaches of the Yangtze river. J. Acta Hydrobiol. Sin..

[32] Zheng Y., Zhang L., Zhou Y., Zhang B. (2017). Vegetation Change and Its Driving Factors in Global Drylands during the Period of 1982–2012. J. Arid Zone Res..

[33] Deng Y., Xiao Y., Tuo Y., He T., Cao J. (2016). Influence of Three Gorges Reservoir on water temperature between Yichang and Jianli. J. Adv. Water Sci..

[34] Peng Q., Liao W., Li C., Yu X. (2012). Impacts of Four Major Chinese Carps’ Natural Reproduction in the Middle Reaches of Changjiang River by Three Gorges Project Since the Impoundment. Adv. Eng. Sci..

[35] Chang T., Gao X., Liu H. (2019). Variation of Runoff in the Yichang Reach of Yangtze River and its Influence to the Chinese Sturgeon Spawning Under the Cascaded Hydropower Operation. Acta Hydrobiol. Sin..

